# Efficacy and safety of interspinous process device compared with alone decompression for lumbar spinal stenosis: A systematic review and meta-analysis

**DOI:** 10.1097/MD.0000000000038370

**Published:** 2024-06-07

**Authors:** Changjiu Zhu, Guiling Xiao

**Affiliations:** a Department of Orthopedics, Sichuan Provincial People’s Hospital, Chengdu, China.

**Keywords:** effect, interspinous process device, lumbar spinal stenosis, meta-analysis

## Abstract

**Study Design:**

Systematic review and meta-analysis.

**Background:**

Interspinous process devices (IPD) were used as a treatment in selected patients with lumbar spinal stenosis (LSS). However, the use of IPD was still debated that it had significantly higher reoperation rates compared to traditional decompression. Therefore, the purpose of the meta-analysis was to evaluate the effectiveness and safety of IPD treatment in comparison to traditional treatment.

**Methods:**

The databases were searched of PubMed, Embase and the Cochrane, Chinese National Knowledge Infrastructure, Chongqing VIP Database and Wan Fang Database up to January 2024. Relevant studies were identified by using specific eligibility criteria and data was extracted and analyzed based on primary and secondary endpoints.

**Results:**

A total of 13 studies were included (5 RCTs and 8 retrospective studies). There was no significant difference of Oswestey Disability Index (ODI) score in the last follow-up (MD = −3.81, 95% CI: −8.91–1.28, *P* = .14). There was significant difference of Visual Analog Scale (VAS) back pain scoring in the last follow-up (MD = −1.59, 95% CI: −3.09–−0.09, *P* = .04), but there existed no significant difference of leg pain in the last follow-up (MD = −2.35, 95% CI: −6.15–1.45, *P* = .23). What’s more, operation time, bleeding loss, total complications and reoperation rate had no significant difference. However, IPD had higher device problems (odds ratio [OR] = 9.00, 95% CI: 2.39–33.91, *P* = .001) and lesser dural tears (OR = 0.32, 95% CI: 0.15–0.67, *P* = .002) compared to traditional decompression.

**Conclusion:**

Although IPD had lower back pain score and lower dural tears compared with traditional decompression, current evidence indicated no superiority for patient-reported outcomes for IPD compared with alone decompression treatment. However, these findings needed to be verified in further by multicenter, double-blind and large sample RCTs.

## 1. Introduction

The annual incidence of lumbar spinal stenosis (LSS) is reported to be 5 cases per 100,000 individuals, significantly surpassing the incidence of cervical spinal stenosis. Additionally, the prevalence of LSS is continuously on the rise.^[1,2]^ Degeneration of the vertebral disc often leads to narrowing of the recess and the intervertebral foramina, which can lead to facet joint hypertrophy and ligamentum flavum hypertrophy.^[3,4]^ Many individuals have different clinical symptoms of low back pain, leg back, and neurogenic claudication, which seriously affects the quality of life for patients.^[4–6]^ In the case of LSS, the traditional decompression treatment still is the “gold standard” when conservative treatment fails.^[7–9]^

Interspinous process devices (IPD) has been gaining popularity, although it is still in the early stages of clinical use.^[10,11]^ The IPD implant can provide a constant distracting force between the spinous processes, leading to what is known as “indirect decompression.”^[12,13]^ And IPD has been shown to limit the extension of the spine, which may help relieve pain or neurogenic claudication.^[14]^ Therefore, IPD has been designed and tested as an alternative option to traditional decompression treatment for LSS.^[15]^

However, the use of IPD was still debated, with recent reports demonstrating a significantly higher reoperation rate, device problems and with lower cost-effectiveness.^[16]^ But other reports have demonstrated that IPD treatment is increasing and effective.^[17,18]^ Therefore, the question of whether adding additional decompression surgery to IPD is more effective compared to traditional decompression is still being debated. We aim to conduct a systematic review and meta-analysis of the current literature to assess the relative benefits and risks of IPD treatment compared with traditional decompression surgery.

## 2. Materials and methods

### 2.1. Study design

This meta-analysis is in accordance with PRISMA statement.^[19]^

### 2.2. Literature retrieval strategy

The following electronic databases were searched up to January 2024 such as PubMed, Embase and the Cochrane, Chinese National Knowledge Infrastructure, Chongqing VIP Database and Wan Fang Database. All RCTs and retrospective studies comparing IPD treatment compared to traditional decompression surgery for the treatment of LSS were collected. The retrieval method adopted the combination of subject words and free words, and English retrieval words and Chinese versions include: ((((((((Interspinous devices[Title/Abstract]) OR (ISD[Title/Abstract])) OR (DLP[Title/Abstract])) OR (X-Stop[Title/Abstract])) OR (Coflex[Title/Abstract])) OR (DIAM[Title/Abstract])) OR (Wallis[Title/Abstract])) OR (Asperius[Title/Abstract])) AND (((lumbar spinal stenosis[Title/Abstract]) OR (LSS[Title/Abstract])) OR (neurogenic intermittent claudication[Title/Abstract])) in addition, the references of the included literature were reviewed to supplement the relevant studies. Ethical approval for meta-analysis was not required because meta-analysis did not involve any subject directly.

### 2.3. Inclusion and exclusion criteria

#### 2.3.1. Inclusion criteria

Studies were eligible for inclusion if they met the following criteria: Study design: RCTs and retrospective studies; availability of adequate raw data for clinical outcomes; Compared IPD treatment with traditional decompression treated patients with LSS; LSS was diagnosed by preoperative imagine; Conservative treatment was not effective and patients with LSS was needed surgical treatment.

#### 2.3.2. Exclusion criteria

Studies were ineligible if they met the following criteria: studies could not extract data studies so that the study could not be analyzed; duplicate reports; other interventions or drug use; relevant outcome indexes were not reported; animal studies, biomechanical studies, duplicate publications, case report, letter, revision, technology note, commentaries, reviews, withdraw trails and meta-analysis.

### 2.4. Data extraction

Two researchers independently read the full text of potential studies that met the inclusion and exclusion criteria. The data were extracted as follows: basic information on the sample included in the study (year of publication, total number of participants, authors, age, gender, and intervention etc); study design type (prospective and retrospective study), study duration, and study observation indicators, etc What’s more, we extracted the following data from each selected study: Oswestey Disability Index (ODI) score, Visual Analog Scale (VAS) pain score, operation time, bleeding loss, complications, reoperation rate, device problems and dural tears. When information was missing, we attempted to contact the primary author via email to seek clarification or exclude the study.

### 2.5. Risk of bias assessment

The risk of bias in the included studies was evaluated using the JADAD’s scale assessment tool for RCTs. The JADAD scale score range from 0 to 5. Studies scoring lower than 3 were considered as low quality.^[20]^ Newcastle–Ottawa Scale for retrospective studies, the studies scored ≥ 6 were considered to be high quality articles.^[21]^ Bias assessments were carried by 2 researchers independent. Any unresolved disagreements between reviewers were resolved through discussion or by evaluation by a third reviewer.

### 2.6. Statistical analysis

The Revman 5.4 software package was used for this meta-analysis. The dichotomous outcomes were reported by odds ratio (OR) with 95% confidence interval (CI) and we reported continuous outcomes for mean difference (MD) with 95% CI. Heterogeneity test was performed on the included study results by chi-square test. If I^2^ ≤ 50%, it indicated that there was no homogeneity among the research results, and a fixed effect model was used. If *P* < .05 and I^2^ > 50%, then, heterogeneity existed among studies, and a random effect model was used. We also performed a sensitivity analysis to identify the resource of the heterogeneity. Publication bias was assessed by the funnel plot.

## 3. Results

### 3.1. Search result

**Figure 1.**
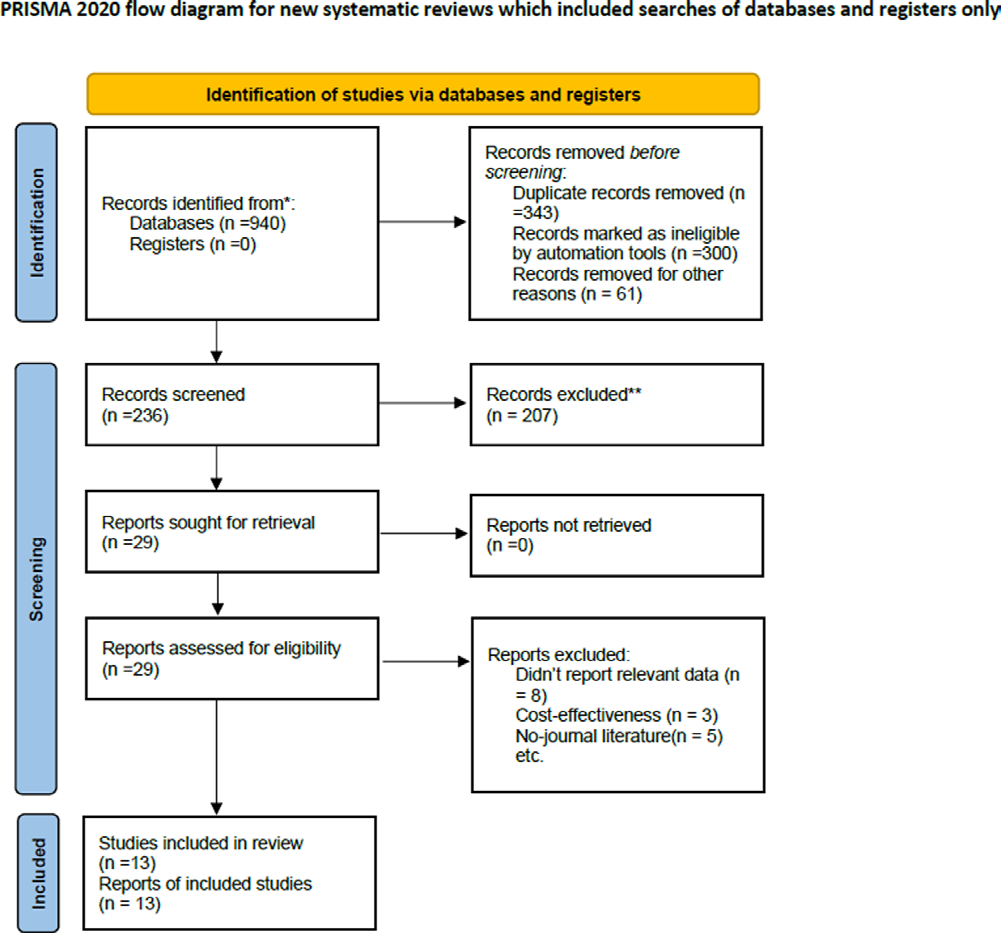
The flowchart of the study.

The initial search yielded 940 records where we excluded 343 records due to the duplication. After examination of the titles, abstracts and full-text articles 13 potentially eligible studies assessed for inclusion criteria. After application of the inclusion criteria, 11 trials published in English and 2 trials published in Chinese were included in this meta-analysis. Figure 1 displays the selection algorithm, numbers of included and excluded studies. All titles, abstracts, and text were dually and independently reviewed by the authors based on the inclusion and exclusion criteria to minimize bias.

### 3.2. Study characteristic

5 RCTs and 8 retrospective studies included 954 patients in this meta-analysis. Among those included studies, 6 trails used Coflex device, 4 trials used DIAM device, 2 trials used Wallis device and 1 trial used In-space device. The main basic characteristics of the included literatures were shown in Table 1.

**Table 1:** Basic characteristics of the included literature.

Name	Year	Age (I/C)	Number of persons (I/C)	Intervention group	Controlled group	Follow-up time (I/C)
Ryu, S.J.^[22]^	2010	69.38/72.31	16/20	DIAM	Decompression	21.43/22.75
Kumar, N.^[23]^	2014	57.9/61.8	22/24	Coflex	Decompression	24/24
Richter, A.^[24]^	2014	68/68	31/31	Coflex	Decompression	24/24
Roder, C.^[25]^	2015	67.6/63	50/50	Coflex	Decompression	9.2/6.9
Schmidt, S.^[17]^	2018	68/68	110/115	Coflx	Decompression	24/24
Wang, S.L.^[26]^	2013	45.6/45.4	26/26	Wallis	Decompression	NA
Du, R.^[27]^	2011	63.80/65.90	16/17	In-Space	Decompression	13.2/13.2
Marsh, G.D.J.^[28]^	2014	59.6/56.4	30/30	Wallis	Decompression	40/40
Galarza, M.^[29]^	2014	38.5/42.5	45/47	DIAM	Decompression	24/24
Holinka, J.^[30]^	2011	73/71	22/28	DIAM	Decompression	44/46
Kim, K.A.^[31]^	2007	51/50	31/31	DIAM	Decompression	12/12
Ghany, W.A.^[32]^	2016	55/51.8	28/25	Coflex	Decompression	12/12
Jack, Z.^[33]^	2021	69/64.2	46/37	Coflex	Decompression	NA

NA: not available.

### 3.3. The bias risk assessment results of the included studies

Retrospective studies used NOS scale to evaluate the risk of bias. The included retrospective studies met most of the quality assessment criteria, and all these studies were scaled as a total score of ≥ 6, indicating a low risk of bias. The detail of information could be seen in Table 2. RCTs were evaluated by the JADAD’s scale. The quality assessment of included studies was shown in Table 3 for details.

**Table 2:** Results of quality assessment using Newcastle–Ottawa scale for cohort studies.

Study selection	Representativeness of the exposed cohort	Selection of the nonexposed cohort	Ascertainment of exposure	Demonstration that outcome of interest was not present at start of study	Comparability of cohorts on the basis of the design or analysis	Assessment of outcome	Follow-up long enough for outcomes to occur	Adequacy of follow-up of cohorts	Quality score
Ryu, S.J. 2010^[22]^	1	1	1	1	1	1	1	1	8
Kumar, N 2014^[23]^	1	1	1	1	1	1	1	1	8
Roder, C. 2015^[25]^	1	1	1	1	1	1	0	1	7
Holinka, J. 2011^[30]^	1	1	1	1	1	1	1	1	8
Kim, K.A. 2007^[31]^	1	1	1	1	1	1	1	1	8
Ghany, W.A. 2016^[32]^	1	1	1	1	1	1	1	1	8
Wang, S.L. 2013^[26]^	1	1	1	1	1	1	1	1	8
Jack, Z. 2021^[33]^	1	1	1	1	0	1	1	1	7

**Table 3:** Results of quality assessment using JADA’s score for RCTs.

	Randmization	Double blinding	Withdrawals and dropouts	Quality score
Richter, A. 2014^[24]^	1	0	1	2
Schmidt, S. 2018^[17]^	2	2	1	5
Du, R. 2011^[27]^	2	0	1	3
Galarza, M. 2014^[29]^	1	1	1	3
Marsh, G.D.J. 2014^[28]^	2	0	1	3

### 3.4. Meta-analysis results

#### 3.4.1. Oswestey disability index

**Figure 2.**
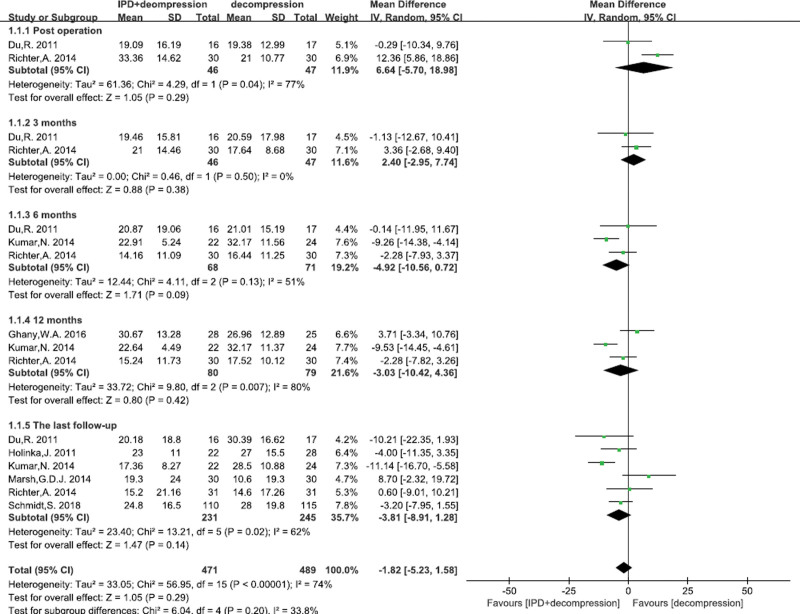
A forest plot showing the ODI score.

A total of 7^[17,23,24,27,28,30,32]^ studies reported the ODI score. There was significant heterogeneity, so random effects model was performed. We failed to find any significant differences between IPD treatment and traditional decompression treatment at post operation (MD = 6.64, 95% CI: −5.70–18.98, *P* = .29; Fig. 2), 3 months (MD = 2.40, 95% CI: −2.95–7.74, *P* = .38; Fig. 2), 6 months (MD = −4.92, 95% CI: −10.56–0.72, *P* = .09; Fig. 2), 12 months (MD = −3.03, 95% CI: −10.42–4.36, *P* = .42; Fig. 2), and at the last follow-up (MD = −3.81, 95% CI: −8.91–1.28, *P* = .14; Fig. 2).

#### 3.4.2. Visual analog scale pain score

**Figure 3.**
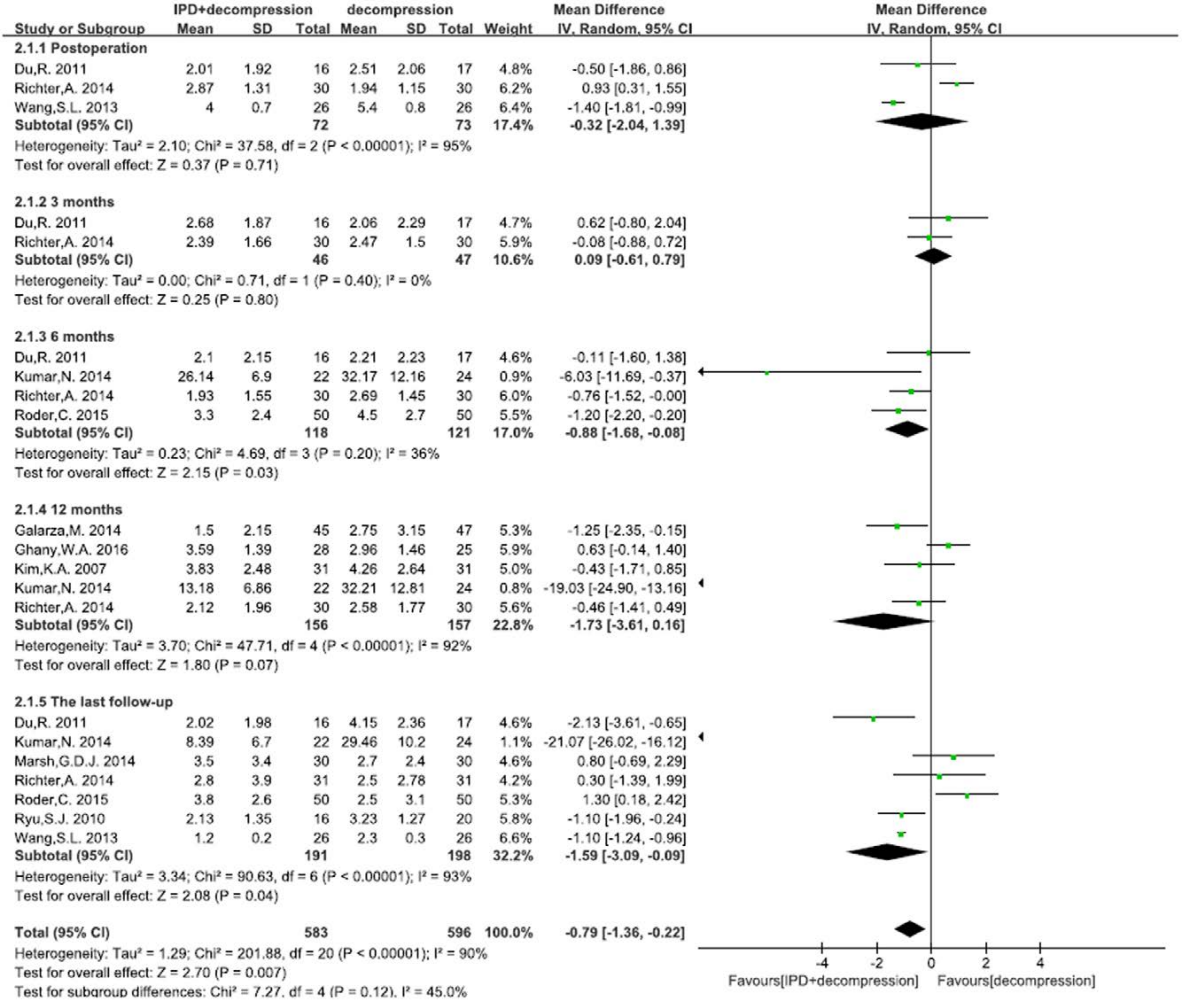
A forest plot showing the VAS score for back pain.

A total of 10^[22–27,29–32]^ studies reported the VAS pain scoring. There was significant heterogeneity of studies, so random effects model was performed. No significant differences in VAS score on back pain were found between 2 groups at post operation (MD = −0.32, 95% CI: −2.04–1.39, *P* = .71; Fig. 3), 3 months (MD = 0.09, 95% CI: −0.61–0.79, *P* = .80; Fig. 3), and 12 months (MD = −1.73, 95% CI: −3.61–0.16, *P* = .07; Fig. 3). However, a significant difference was found at 6 months (MD = −0.88, 95% CI: −1.68–−0.08, *P* = .03; Fig. 3), and at the last follow-up (MD = −1.59, 95% CI: −3.09–−0.09, *P* = .04; Fig. 3). However, we found that the back pain score was lower with the prolongation of follow-up time.

**Figure 4.**
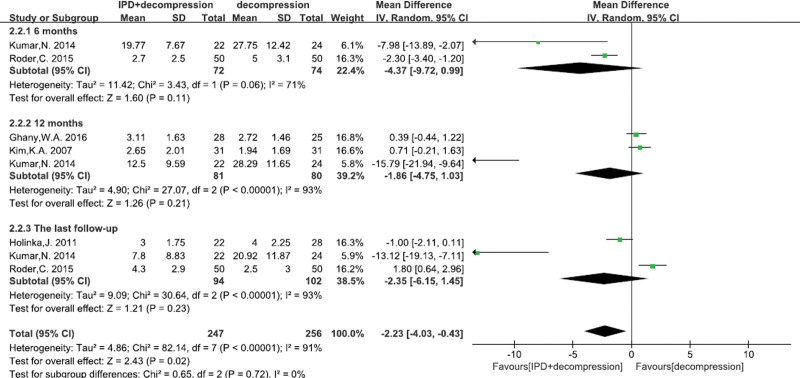
A forest plot showing the VAS score for leg pain.

What’s more, no significant difference on leg pain were found between 2 groups at 6 months (MD = −4.37, 95% CI: −9.72–0.99, *P* = .11; Fig. 4), 12 months (MD = −1.86, 95% CI: −4.75–1.03, *P* = .21; Fig. 4), and at the last follow-up (MD = −2.35, 95% CI: −6.15–1.45, *P* = .23; Fig. 4).

#### 3.4.3. Operation time

**Figure 5.**
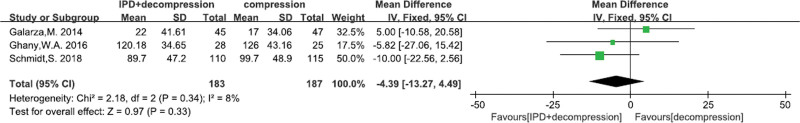
A forest plot showing the hospital time.

A total of 3^[17,29,32]^ studies reported the operation time. There was no significant heterogeneity (*P* = .34, I^2^ = 8%). Fixed effects model was performed. There was no significant difference between IPD treatment and traditional decompression treatment (MD = −4.39 min, 95% CI: −13.27–4.49, *P* = .33; Fig. 5).

#### 3.4.4. Blood loss

**Figure 6.**
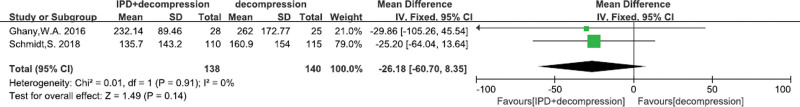
A forest plot showing the blood loss.

A total of 2^[17,32]^ studies reported the blood loss. There was no significant heterogeneity (*P* = .91, I^2^ = 0%). Fixed effects model was performed. There was no significant difference between IPD treatment and traditional decompression treatment (MD = −26.18 min, 95% CI: −60.70–−8.35, *P* = .14; Fig. 6).

#### 3.4.5. Total complications

**Figure 7.**
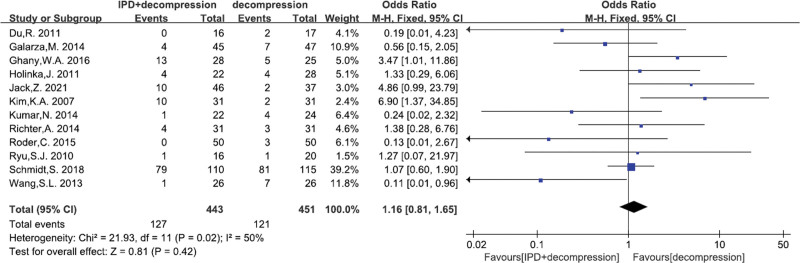
A forest plot showing the total complications.

A total of 12^[17,22–27,29–33]^ studies reported the Total complications. There was no significant heterogeneity (*P* = .02, I^2^ = 50%). Fixed effects model was performed. There was no significant difference between IPD treatment and traditional decompression treatment (MD = 1.16, 95% CI: 0.81–1.65, *P* = .42; Fig. 7).

#### 3.4.6. Reoperation rate

**Figure 8.**
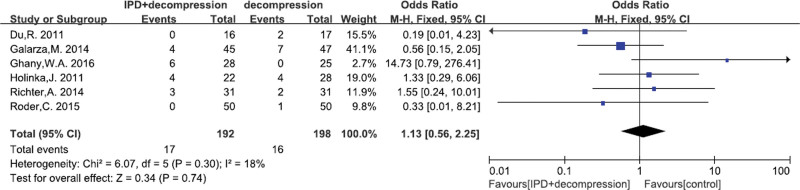
A forest plot showing the reoperation rate.

A total of 6^[24,25,27,29,30,32]^ studies reported the reoperation rate. There was no significant heterogeneity (*P* = .30, I^2^ = 18%). Fixed effects model was performed. There was no significant difference between IPD treatment and traditional decompression treatment (MD = 1.13, 95% CI: 0.56–2.25, *P* = .74; Fig. 8).

#### 3.4.7. Device problems

**Figure 9.**
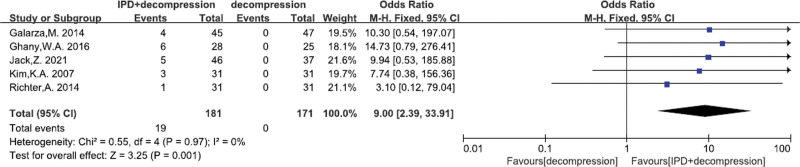
A forest plot showing the device problems.

A total of 5^[24,29,31–33]^ studies reported the device problems. There was no significant heterogeneity (*P* = .97, I^2^ = 0%). Fixed effects model was performed. In terms of device problems significantly higher were obtained in the IPD treatment (MD = 9.00, 95% CI: 2.39–33.91, *P* = .001; Fig. 9).

#### 3.4.8. Dural tears

**Figure 10.**
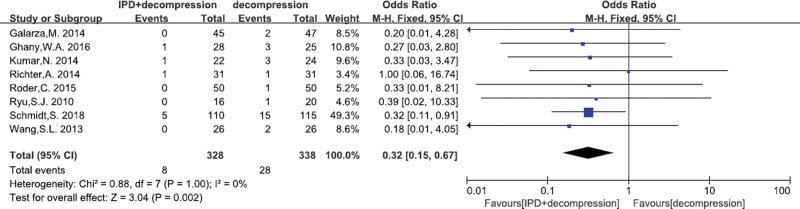
A forest plot showing the dural tears.

A total of 8^[17,22–26,29,32]^ studies reported the dural tears. There was no significant heterogeneity (*P* = 1.0, I^2^ = 0%). Fixed effects model was performed. In terms of dural tears significantly lower were obtained in the IPD treatment (MD = 0.32, 95% CI: 0.15–0.67, *P* = .002; Fig. 10).

#### 3.4.9. Anterior disc height

**Figure 11.**
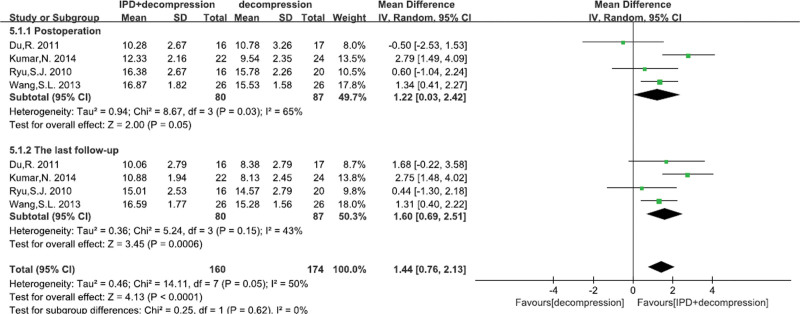
A forest plot showing the anterior disc height.

A total of 4^[22,23,26,27]^ studies reported the anterior disc height. There was significant heterogeneity, so random effects model was performed. We found significant differences between IPD treatment and traditional decompression treatment at post operation (MD = 1.22 mm, 95% CI: 0.03–2.42, *P* = .05; Fig. 11), and at last follow-up (MD = 1.60 mm, 95% CI: 0.69–2.51, *P* = .0006; Fig. 11).

#### 3.4.10. Anterior disc height

**Figure 12.**
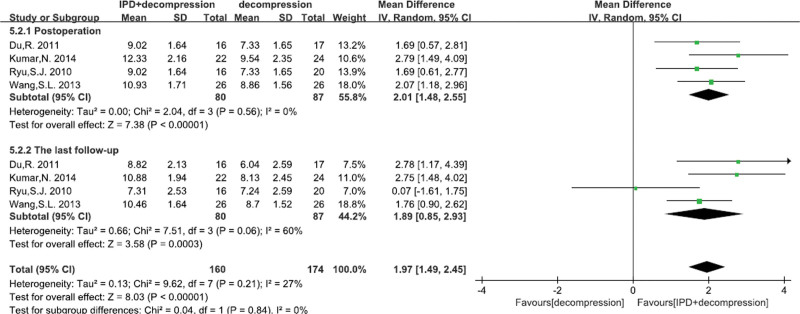
A forest plot showing the posterior disc height.

A total of 4^[22,23,26,27]^ studies reported the posterior disc height. There was significant heterogeneity, so fixed effects model was performed. We found significant differences between IPD treatment and traditional decompression treatment at post operation (MD = 2.01 mm, 95% CI: 1.48–2.55, *P* < .00001; Fig. 12), and at last follow-up (MD = 1.97 mm, 95% CI: 0.85–2.93, *P* = .0003; Fig. 12).

### 3.5. Publication bias

**Figure 13.**
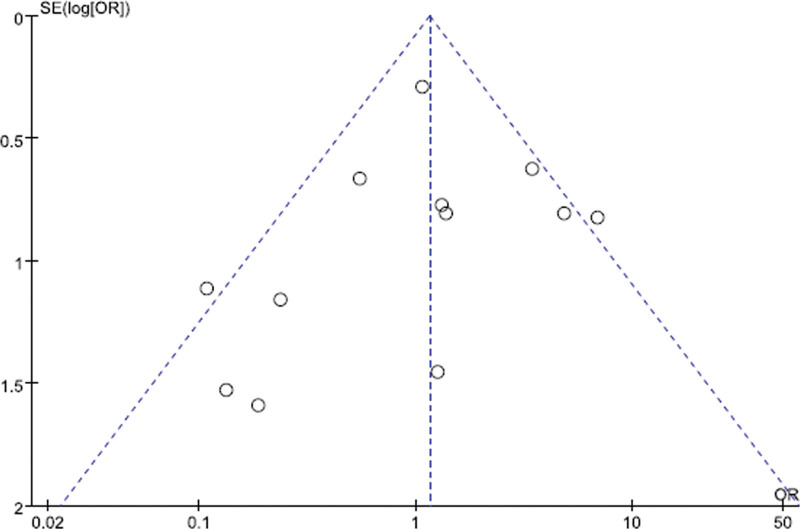
A funnel plot showing publication bias.

The funnel plot was used to evaluate the publication bias of studies. For studies in complications, the funnel plot was not symmetry (Fig. 13). It indicated the possibility of publication bias might exist.

## 4. Discuss

LSS was a degenerative disease of vertebra that can compress corresponding spinal segments or nerves, causing low back pain, leg pain, neurogenic intermittent claudication.^[34]^ The optimal treatment is generally considered to be surgical intervention, with studies reporting that decompression treatment alone is the “gold standard” in treating LSS. IPD was invented by Abbott Spine in 1986 and has been widely used in clinical practice. Recently, 3 RCTs from the Netherlands, Sweden, and Norway compared IPD with traditional decompression, and found that the IPD group had a higher reoperation rate. However, both IPD and decompression treatment alone showed similar clinical effects in treating LSS.^[35–37]^ However, some studies showed the safety and efficacy of IPD treatment compared with traditional decompression treatment.^[25,28,29]^ However, the recommendations or guidelines had not been established regarding which type of surgical treatment was preferable to improve postoperative clinical outcomes. Hence, we tried to analysis whether IPD treatment could achieve better clinical outcomes compared with alone decompression treatment.

ODI was an important functional activity and motion preservation indicator to measure degree of disability and estimate quality of life in patients.^[38]^ Our meta-analysis results demonstrated IPD treatment had no significant difference for ODI, and the results of studies included in our meta-analysis showed high heterogeneity. Richter et al^[24]^ reported that IPD treatment and alone decompression treatment could improve clinical outcomes from baseline, but the ODI in 2-year follow-up had no significant difference between IPD treatment and alone decompression treatment. Similar clinical results were also demonstrated by Marsh et al^[28]^ What’s more, a prospective, randomized, multicenter study with 2-year follow-up also demonstrated that a higher percentage of patients in the IPD treatment group achieved ODI success with improvement > 15 points, however, the results were not statistical difference.^[17]^ Although traditional decompression surgery, as a “golden standard,” was effective to treat LSS,^[39,40]^ our meta-analysis demonstrated that traditional decompression surgery was not inferior to IPD treatment in terms of ODI.

VAS was a useful indicator to evaluate patient’s functional recovery for LSS.^[41]^ It is generally agreed that degenerative spinal stenosis with low back pain and leg pain should be treated with nerve root decompression surgery.^[42,43]^ And the decompression with less preservation of the posterior elements could induce instability or recurrent stenosis, which affects the clinical outcome. Some studies demonstrated that the most frequently performed procedure was posterior decompression surgery, with the primary aim of relieving leg pain symptoms. Although low back pain also often be reduced after surgery, decompression was generally thought to yield worse results for low back pain than for leg pain. Hence, the posterior decompression surgery was suggested for patients with leg predominant symptoms.^[44,45]^ Our meta-analysis demonstrated that the leg pain had no significant difference between IPD treatment and alone decompression surgery at different time-points of follow-up (6, 12 mo and the last follow-up). We considered that patients in both groups received traditional decompression surgery, which effectively alleviated leg pain by nerve root decompression. However, we pooled data analysis demonstrated that IPD treatment was better to traditional decompression surgery in terms of low back pain, especially at the end of the follow-up. Yuan et al^[46]^ reported that the IPD could regulate range of motion of the superior adjacent segment by indirect decompression to relieve low back pain. And a prospective, randomized, controlled study demonstrated that IPD treatment was more excellent than alone decompression treatment (*P* < .01) by using modified MacNab criteria for low back pain.^[29]^ Although no significant difference was found between 2 groups in the leg pain, IPD treatment was better than alone decompression treatment for low back pain.

Some studies suggested that IPD group had higher reoperation rate and complication rate.^[16,47,48]^ However, our pooled data analysis demonstrated that there was no significant difference in operation time, bleeding loss, complication rate and reoperation rate between IPD treatment and traditional decompression treatment. What’s more, we found that alone decompression surgery had more dural tears compared with IPD treatment. And some studies suggested that alone decompression treatment can result in hemorrhage, dural lesion, wound infection, restenosis of vertebral canal and spinal or nerve root injury etc^[49,50]^ However, our meta-analysis found that device problems rate was higher with the prolongation of follow-up time in IPD treatment. IPD, as a implants, can also induce some sever adverse events, such as spinous process fracture and spinal or nerve root injury.^[50,51]^ Finally, we also found that IPD treatment had higher posterior disc height and anterior disc height compared with alone compression. Because radiologically, IPD would increase the height of the posterior disc, prevent or reduce the degree of spondylolisthesis, and correct the lordosis with kyphosis.^[22,23]^ Hence, our results demonstrated that IPD treatment was an alternative treatment for LSS compared to traditional decompression surgery.

## 5. Current limitation

However, there are also few disadvantages and limitations for our study: Some pooled results from included studies were strongly subjective, which may influence the results due to the different experience from doctors. Most of the included studies were retrospective studies, which mostly affected the results of the experiments. The IPD devices used in these studies were different types and they conducted in different regions. This difference in follow-up time is an important source of heterogeneity and the follow-up duration was too short in some studies, which may hide some outcomes. Inclusion and exclusion criteria for some studies are different. Therefore, physicians around the world should interpret our results with cautions when applying them in clinical practice.

## 6. Conclusion

Current evidence indicated no superiority for patient-reported outcomes for IPD treatment compared with alone decompression treatment. Although IPD treatment had lower back pain score and lower dural tears compared with traditional decompression surgery, there are no significant difference in other clinical outcomes. Based on these results, we suggested IPD treatment and alone decompression surgery were acceptable strategies for LSS, and the risks, indications, costs should be carefully taken into account. More important, larger sample size studies and longer follow-up to assess the IPD treatment were needed.

## Author contributions

**Conceptualization:** Changjiu Zhu, Guiling Xiao.

**Data curation:** Changjiu Zhu, Guiling Xiao.

**Formal analysis:** Changjiu Zhu, Guiling Xiao.

**Funding acquisition:** Guiling Xiao.

**Methodology:** Changjiu Zhu, Guiling Xiao.

**Project administration:** Changjiu Zhu.

**Writing – original draft:** Changjiu Zhu, Guiling Xiao.

**Writing – review & editing:** Changjiu Zhu, Guiling Xiao.
